# Does digital inclusive finance promote carbon emission reduction of enterprises

**DOI:** 10.1371/journal.pone.0302826

**Published:** 2024-07-01

**Authors:** Yu Peng, Ying Qiu, You Li, Xinwan Peng

**Affiliations:** 1 Department of Business, Macau University of Science and Technology, Macau, P. R. China; 2 Department of Economics and Management, Beijing Forestry University, Beijing, P. R. China; 3 Department of Economics, Jiangxi University of Finance and Economics, Nanchang, Jiangxi Province, P. R. China; Guilin University of Aerospace Technology, CHINA

## Abstract

Can the information technology revolution lead to carbon emission reduction for firms? This study extends the limited evidence in the literature and investigate the role and mechanism of digital inclusive finance on enterprises’ carbon emissions using panel data of 247 prefectural-level cities and 6019 industrial enterprises in China. Our findings indicate that digital inclusive finance can promote enterprise carbon emission reduction, and this effect remains significant after the instrumental variable estimation test. The effect has regional heterogeneity and the development of digital inclusive finance in the area east of Hu Huanyong line has a significant impact on reducing enterprise carbon emission. The role of digital inclusive finance is heterogeneous in enterprise ownership, with a remarkable effect in non-state-owned enterprises. Sub-dimension analysis indicates that the breadth of coverage, depth of use, and degree of digitalization of digital inclusive finance have differential effects on reducing enterprise carbon emissions. The stepwise regression method shows that the impact of digital inclusive finance on enterprise carbon emissions can be passed through effect of technological progress, environmental protection investment and financing constrain. This study has significant reference value for evaluating the impact of financial inclusion and policy implications in formulating differentiated strategies for achieving carbon emission reduction efficiency in enterprises.

## 1.Introduction

In recent years, China has shifted its focus towards sustainable development as it enters a "new normal" phase in its economy. This has led to an increased emphasis on carbon emission reduction, which has become a central research area. Numerous studies have explored various strategies for reducing carbon emissions, such as technological improvements, energy structure adjustments, and increasing foreign investment [1–5]. These studies have provided valuable insights for policy formulation in China to achieve its "double carbon" targets.

As the information technology revolution continues to advance, digital finance has emerged as a significant driving force for economic development. Digital inclusive finance, defined as the utilization of digital technology to provide financial services to populations traditionally excluded from the formal financial system [5,6]., has garnered global attention. Many countries and organizations, including China, have prioritized the growth and adoption of digital inclusive finance. In this context, the Chinese government has implemented a series of policies to foster digital finance development, such as creating a national digital currency and establishing a regulatory sandbox for fintech companies. These efforts aim to promote financial inclusion and support the growth of small and medium-sized enterprises (SMEs) and other underserved populations.

This study aims to elucidate the positive effect of digital inclusive finance on promoting enterprise carbon emission reduction. Furthermore, it underscores that the impact of digital inclusive finance on enterprise carbon emission reduction is regionally heterogeneous, enterprise ownership heterogeneous. Moreover, it acknowledges that the sub-dimensions of digital inclusive finance both in terms of coverage breadth, usage depth, and digitalization can exert distinct effects on reducing enterprise carbon emissions; This paper also corroborates the underlying mechanisms of the effect of digital inclusive finance on enterprises’ carbon emissions—effect of technological progress and environmental protection investment.

To demonstrate the pivotal role of digital inclusive finance on enterprises’ carbon emission reduction, firstly, the article employs instrumental variables to mitigate the potential endogenous issues brought by two-way causality, and the results affirm the significance of digital inclusive finance on enterprises’ carbon emission reduction even after replacing instrumental variables, which further authenticates the reliability of the conclusion. Secondly, through the heterogeneity test, we verify the differences in the role of digital inclusive finance on enterprises’ carbon emission reduction based on geographical dimensions distinction, enterprise ownership distinction, and the division of different sub-dimensions of digital inclusive finance. The above findings further corroborate the role of digital inclusive finance on enterprises’ carbon emission reduction. Additionally, we test the mechanism of technology into this effect. The results show that digital inclusive finance can promote the progress of production technology, thereby facilitating carbon emission reduction in enterprises. Finally, we investigate the mechanism of environmental protection investment. Digital inclusive finance can significantly increase enterprises’ environmental protection investment, and enterprises’ environmental protection investment enhance enterprises’ ability to treat pollutants, implying that digital inclusive finance may promote enterprises’ carbon emission reduction by increasing enterprises’ environmental protection investment.

The current study seeks to explore the impact of digital inclusive finance on promoting carbon emission reduction in enterprises. Specifically, the research questions addressed in this paper include:

1. Does digital inclusive finance have a positive effect on enterprise carbon emission reduction?
2. Are the impacts of digital inclusive finance on carbon emission reduction regionally and enterprise ownership heterogeneous?
3. How do the sub-dimensions of digital inclusive finance, in terms of coverage breadth, usage depth, and digitalization, differentially impact enterprise carbon emissions?

To answer these questions, the study employs various methods such as instrumental variables, heterogeneity tests, and mechanism tests. The results indicate that digital inclusive finance has a positive effect on carbon emission reduction, with regional and enterprise ownership heterogeneity. The findings also reveal that the sub-dimensions of digital inclusive finance have differential effects on carbon emission reduction.

Furthermore, the study identifies two mechanisms through which digital inclusive finance influences carbon emissions: technological progress and environmental protection investment. The results suggest that digital inclusive finance promotes technological advancements, leading to carbon emission reduction in enterprises. Additionally, digital inclusive finance significantly increases enterprises’ environmental protection investment, which in turn improves their ability to treat pollutants and reduce carbon emissions.

This study provides a comprehensive analysis of the role of digital inclusive finance in promoting enterprise carbon emission reduction. The findings contribute to the ongoing debate on sustainable development and carbon emissions while offering valuable insights for policymakers and stakeholders in the digital finance domain.

## 2.Literature review

This study contributes significantly to the existing literature in three key ways. First, it enriches the current body of research on digital inclusive finance which predominantly concentrates on its economic and social promotion role, with some studies addressing its impact on carbon reduction: the study on digital inclusive finance and urban-rural income gap has explored how the incorporation of digital technology can improve the construction of digital infrastructure network, pinpoint financial needs, reduce the cost of financial services, and improve their availability, thus bridging the urban-rural income gap [6,7]; and the positive impact of digital inclusive financial services on household consumption [8], highlighting digital inclusive finance’s potential to alleviate entrepreneurs’ financing constraints, and Gao,Y et al. (2022) analyzes the positive relationship between digital inclusive finance and entrepreneurship based on this mechanism [9]; Beyond this, certian studies have explored the carbon reduction effect of digital inclusive finance [10–14].

Second, we enrich the current study on carbon dioxide emission of enterprises. Enterprises represent major contributors to carbon dioxide emissions, thus their production behavior will have a critical impact on the achievement of the "double carbon" target in China. We notice that existing literature on enterprises’ carbon emission reduction research largely falls into two main categories: policy-based research and financing-based research.

Policy-based research explores various strategies to reduce carbon emissions(i.e. [15–22]). For example, Li et al. (2022) utilize structural decomposition analysis (SDA) and computable general equilibrium (CGE) models to demonstrate that tax rates can contribute to carbon emission reduction by optimizing energy consumption structures [15]. Similarly, Bradford et al. (2008) discuss potential policy options for governments to achieve energy efficiency and emission reduction in SMEs [16].

Financing-based research, on the other hand, examines the role of different financing methods in carbon emission reduction. Jiang et al. (2015) found that foreign-owned enterprises are more likely to adopt advanced carbon emission technologies compared to state-owned enterprises [23]. Tang et al. (2022) studied the positive impact of financial deepening on carbon emission reduction, with green innovation acting as a mechanism for this reduction [24]. Yu et al. (2022) investigated the negative impact of financing constraints on emission reduction [25,26],. However, these financing methods generally have high thresholds and insufficient inclusiveness.

Current research on the role of digital inclusive finance in enterprises primarily focuses on alleviating financing pressures [27,28], reducing costs, and promoting entrepreneurship, innovation, and performance improvement [29,30]. There is limited research on the long-term impact of digital inclusive finance on carbon emission reduction, such as its potential role in promoting green innovation. Our study aims to further investigate whether digital inclusive finance can promote enterprise carbon reduction through the mechanism of green innovation, contributing to a more comprehensive understanding of the relationship between digital inclusive finance and carbon emission reduction in enterprises.

Third, we focus on enterprises level, a pivotal economics entity in China economy, providing new micro-level evidence to clarify the carbon emission reduction impact of digital inclusive fiannce by examining the relationship between these two factors and identifying the heterogeneity in enterprises’ carbon emission reduction. Previous studies have explored the impact of digital finance on carbon emission reduction from both macro and micro perspectives, while this research supplements the research on digital inclusive finance at the micro-level using the enterprises level data.

Several studies in academia have explored the role of digital inclusive finance in carbon emission reduction from both macro and micro perspectives. For instance, from macro perspective, Wang et al. (2022) analyzed the positive impact of digital inclusive finance on urban carbon emission reduction, demonstrating the spatial spillover effect [11,31]; Lee et al. (2022) argued that digital inclusive finance reduces carbon emissions by optimizing industrial structure and promoting green technology innovation[12]; Zheng et al. (2022) found that digital inclusive finance reduces carbon dioxide emissions by decreasing per capita energy consumption and increasing per unit of capital GDP using provincial panel data in China [32].

From a micro perspective, Sun et al. (2022) demonstrated the carbon reduction effect of digital inclusive finance in agriculture and its spillover effect [14]; Xu et al. (2022) used the Super-EBM model to study the supporting role of digital inclusive finance for marine carbon sink fisheries and its influencing factors, highlighting that enhancing marine carbon sink capacity is beneficial to CO2 reduction [13,33]. Some studies, based on samples of publicly listed enterprises, encounter issues of sample selection bias, while this research ignores this consideration and uses data from industrial enterprises to supplement the research on digital inclusive finance at the micro-level [34,35].

We integrate digital inclusive finance and enterprise carbon emission reduction within the same framework, using data from 6019 above-scale industrial enterprises to evaluate the impact of digital inclusive finance on carbon emission reduction. To provide a comprehensive overview, Our paper presents a novel approach to the existing literature by constructing an analytical framework that connects digital inclusive finance, financial service availability, optimization of enterprise innovation inputs, market competition and integration, energy efficiency, and carbon emission reduction. We investigate the impact of digital inclusive finance on promoting enterprise carbon emission reduction, revealing a positive effect. Furthermore, the study demonstrates that the influence of digital inclusive finance on carbon emission reduction varies depending on regional heterogeneity and enterprise ownership types. Consequently, our paper offers fresh insights into the role of digital inclusive finance in fostering sustainable development and emphasizes the importance of considering regional and ownership differences when designing policies to support sustainable development through digital inclusive finance.

The remaining of the paper is structured as follows: the second section presents the theoretical analysis and research hypotheses; the third section constructs the research model, and describes the variables and data; the fourth section reports and discusses the empirical results; the final section concludes the paper with policy implications.

## 3.Theoretical analysis and research hypothesis

### 3.1 Digital inclusive finance promotes carbon emission reduction of enterprises

Prior literature often point to financing constraint as the primary hurdle that inhibit the enterprises’ technological innovation in energy conservation and environmental protection, leading to an increase in pollutant emissions and thus hindering the green transformation of enterprises’ production mode [19]. Such friction can be mitigated as the advantage of digital inclusive finance is that it can meet the demands of different scales of enterprises for financial services to the greatest extent, and it promotes enterprises to achieve carbon emission reduction through the following mechanism: digital inclusive finance——enhancing the availability of financial services——promoting innovation, market competition and integration——efficient use of energy——enterprises achieving carbon emission reduction. First, due to the risk aversion of commercial banks, the main providers of traditional financial services, most of the traditional financial services flow to state-owned enterprises (or large and medium-sized enterprises) and high-net-worth enterprises, which limits the access of Small and medium-sized enterprises (SMEs) to financial services [36]. Digital inclusive finance uses digital technologies such as Internet, cloud computing and blockchain to enhance the coverage, depth of use and digitalization of financial services, so that more enterprises can enjoy more inclusive, efficient and convenient financial services, which makes financial services to achieve the "long-tail effect" [37,38], i.e. overcoming the exclusion of SMEs from accessing financial services in the context of traditional finance. In other words, it overcomes the exclusion of SMEs from access to financial services in the process of traditional financial services. Secondly, competition for financial resources is forcing enterprises to adopt efficient and clean production technology. Accordingly, hypothesis 1 is proposed: digital inclusive finance has promoting effect on enterprises’ carbon emission reduction behaviors.

### 3.2 Heterogeneity of digital inclusive finance in promoting carbon emission reduction of enterprises

The impact of digital finance on carbon emission reduction can be heterogeneous may vary across regions due to differences in infrastructure, regulatory frameworks, and financial systems. Due to the difference in financial development of different areas, the effect of digital inclusive finance on carbon emission reduction of enterprises has regional heterogeneity. The Hu Huanyong Line is a geographical boundary that distinguishes between humanities and nature, the financial development in the eastern and western regions of Hu Huanyong Line has formed a different situation under the long-term influence of humanities and natural environment [39]. Existing studies show that there is an unbalanced development between regions in China’s economy, and a large number of economic indicators show the difference between the development of eastern and western regions [32]. Since a large number of digital inclusive finance enterprises’ headquarters are concentrated in Hangzhou, the level of development of digital inclusive finance in Hangzhou is in the leading position nationwide, so the distance from the city to Hangzhou is related to the level of development of digital inclusive finance in the city [40], and the area east of the Hu Huanyong line is close to Hangzhou, with a higher level of development of digital inclusive finance and a more obvious effect on carbon emission reduction of enterprises; the area west of the Huanyong line is The area to the west of the Hu Huanyong line is far from Hangzhou, and the development level of digital inclusive finance is low and has a limited effect on carbon emission reduction of enterprises [41]. Accordingly, hypothesis 2 is proposed: the carbon emission reduction effect of digital inclusive finance has regional heterogeneity.

Additionally, the effectiveness of digital finance in reducing carbon emissions may depend on the specific characteristics of firms as pointed out by [42]. Enterprises are divided into state-owned enterprises and non-state-owned enterprises based on their holding status. For example, non-state-owned firms may be better positioned to obtain gains from using digital finance to reduce their carbon footprint, while state-owned firms may face marginal benefits in adopting these technologies. According to Qian, H. et al. (2020),Commercial banks, the main providers of traditional financial services, have a preference for profit maximization and risk avoidance, thus most traditional financial services flow to state-owned enterprises and high-net-worth enterprises, which makes the difference between state-owned enterprises and other non-state-owned enterprises in terms of access to financial services, and traditional finance has provided more adequate financial support for state-owned enterprises [43], and the diminishing marginal benefits of digital inclusive finance for SOEs make the role of digital inclusive finance for such enterprises limited, while non-SOEs, which are "left out" by traditional finance, rely more on obtaining financial services from digital inclusive finance, and the impact of digital inclusive finance on non-SOEs is more obvious. This makes a difference in the degree of impact of digital inclusive finance on enterprises’ carbon emission. Therefor we propose hypothesis 3: The carbon emission reduction effect of digital inclusive finance has enterprises’ ownership heterogeneity according to state-owned and non-state-owned enterprises.

There are sub-dimensional differences in the role of digital inclusive finance in enterprises’ carbon emission reduction [32]. Combining the new situation and new features of digital finance development, the Digital Inclusive Finance Research Center of Peking University constructs an indicator system of digital inclusive finance development from three sub-dimensions: breadth of coverage, depth of use and degree of digitalization. The breadth of coverage is measured by the indicator account coverage rate, which is related to the popularity of digital inclusive financial services; the depth of use refers to the diversity of financial services and products, considering whether the financial services can meet the diversified financial needs of consumers; the degree of digitization is related to whether financial entities can use digital information technology to provide efficient and convenient financial services to target groups at a lower cost. In this paper, three sub-dimensions of digital inclusive finance of Peking University are used for analysis to examine the role of the breadth of coverage, depth of use and degree of digitalization of digital inclusive finance in relation to carbon emission reduction of enterprises. Accordingly, hypothesis 4 is proposed: the three sub-dimensions of digital inclusive finance have different effects on the carbon emission reduction of enterprises.

### 3.3 Analysis of the mechanism of digital inclusive finance affecting enterprises’ carbon emission reduction

#### 3.3.1 Technological progress effect

High-quality innovation, which depends heavily on corporate innovation quality, is the essential way to promote corporate sustainable development in China. Previous studies document that fiscal decentralization significantly improves corporate innovation quality in China [44,45].

While digital inclusive finance is a good tool to reduce the financial barrier and promote resources mobility, one of the key channels through which digital inclusive finance can impact a firm’s carbon emissions is by facilitating the adoption of advanced production technologies, which is an important way for enterprises to improve their production efficiency. Digital inclusive finance can enable firms, particularly small and medium-sized enterprises (SMEs), to access financing options that were previously unavailable or unaffordable. With improved access to capital, these firms can invest in advanced technologies that can help them reduce their carbon footprint, such as renewable energy systems, energy-efficient equipment, and waste reduction solutions. Admittedly, in the short term, investing in improving production technology does not have a significant positive impact on the green production of enterprises, as the initial investment costs are shared, the green performance of enterprises will gradually improve significantly; this a conflicting and complementary goal paradox between the short-term and long-term green performance of enterprises [46].

In addition, digital finance platforms can serve as hubs for information and knowledge sharing, connecting firms with experts, resources, and best practices related to sustainable technology adoption. The higher the level of technology, the more it can promote the utilization of input factors and the transformation of innovative achievements, thus reducing enterprises’ carbon emissions [47]. Technological advances provide the preparation conditions for enterprises to use cleaner energy for production, which helps to optimize the energy consumption structure and reduce enterprises’ carbon emissions [15]. The development of digital inclusive finance lowers the financing threshold and broadens the financing channels and enhances innovation and R&D, information and knowledge sharing for enterprises, who adopt advanced production technologies for cleaner production and more efficient use of resources, thus improving their carbon emission reduction. We propose hypothesis 5: the impact of digital inclusive finance on enterprises’ carbon emissions can be realized through the effect of technological progress.

#### 3.3.2 Environmental protection investment effect

Digital inclusive finance can also enable firms to have easier access to green finance and make environmental protection investment, which has been shown to be crucial for achieving emission reduction targets [48]. According to Jiang H. (2004), environmental protection investment can be divided into three categories: investment in protecting and improving the ecological environment, investment in environmental management and science and technology, and investment in pollution control, of which pollution control investment is the main part of environmental protection investment [49]. Making investments in technology and infrastructure that reduce emissions can lead to significant reductions in greenhouse gas emissions and other harmful pollutants. Although environmental protection investment will increase the production and operation cost of enterprises in the short term, environmental protection investment can offset the cost of environmental regulation in the long term [50]. At the same time, the enterprise’s pollution control investment can help improve the enterprise’s ability to treat pollutants, thus promoting the enterprise’s carbon emission reduction and practicing low-carbon green sustainable development.

Due to the limitations of traditional finance, people in less developed regions don’t have full access to financial resources due to the objective factors such as geographical conditions and accessing costs [51]. Along with this, the problem is that many enterprises in China have insufficient total investment in environmental pollution control investment and financing, unreasonable investment and financing structure, insufficient innovation in investment and financing mechanisms, and lack of diversity in investment and financing channels [52]. Especially for enterprises that cannot fully enjoy traditional financial services, the lack of investment in pollution management realization caused by financing difficulties hinders enterprises’ production of carbon emission reduction. By leveraging digital platforms and innovative financial products, these firms can gain better access to funding opportunities, enabling them to invest in all three categories of green technologies, renewable energy, and sustainable practices. Hypothesis 6 is proposed: digital inclusive finance can promote enterprises’ carbon emission reduction through increasing enterprises environmental protection investment.

#### 3.3.3 Financing constrain effect

Existing research frequently highlights financing constraints as a major obstacle hindering technological innovation in energy conservation and environmental protection within enterprises and thus affect the increase in enterprise pollutant emissions intensity [25].

According to Zou Wei and Ling Jianghuai (2018), inclusive finance aims to progressively provide essential services to individuals excluded from financial services due to the limitations of traditional finance [51]. Digital inclusive finance, as an extension of inclusive finance, utilizes data and innovative financial products to reduce information asymmetry between capital supply and demand.Digital inclusive finance can leverage the benefits of "low cost, rapid speed, and wide coverage" by utilizing various scenarios and data. This approach aims to lower the threshold and service costs of financial services, enhance the financing environment for SMEs, and provide more efficient support to inclusive financial entities [28,53].

In a situation where enterprises experience heightened financing constraints, they might encounter a shortage of cash flow to sustain research and development (R&D) activities, consequently impeding innovation and technological advancements. Digital inclusive finance plays a crucial role in alleviating these financing constraints for enterprises, fostering a more conducive and favorable environment for their technological innovation initiatives and environmental investment activities,as a result, promoting the enterprise’s carbon emission reduction [25].

Hypothesis 7 is proposed: digital inclusive finance can promote enterprises’ carbon emission reduction through decreasing enterprises financing constrain.

## 4 Research design

### 4.1 Model setting

#### 4.1.1 Baseline regression

Our theoretical analysis indicates that a negative relationship exists between digital inclusive finance and enterprise carbon emissions. To formally quantify the effect of digital inclusive finance, we use panel regression to analyze the firm-level carbon emission intensity on the digital inclusive finance score and control variables, with a rich set of fixed effects. The following econometric model is proposed as follows:

$$\ln E_{it}=\alpha_{0}+\alpha_{1}{DIFI}_{it}+\alpha_{2}{Control}_{it}+\lambda_{t}+\mu_{i}+\varepsilon_{it}$$ (1)

In Eq (1), *i* refers to the enterprise and *t* is the year; *E*_*it*_ is the carbon emission intensity of enterprise *i* in year *t*; *DIFI*_*it*_ is the level of development of digital inclusive finance in the corresponding city of enterprise *i* in year *t*; *Control*_*it*_ is the control variable, including the level of economic development (ln *GDP*), employee income (ln *wage*), industrial structure (*STR*), total enterprise output(ln *output*), and enterprise profitability (*roa*); *λ*_*t*_ is the individual fixed effect, *μ*_*i*_ is the time fixed effect, and *ε*_*it*_ is the random error term. The coefficient *α*_1_ is supposed to be negative if the development of digital inclusive finance has effect on reducing enterprises’ carbon emissions.

### 4.2 Variable selection

#### 4.2.1 Response variable: Enterprise carbon emissions (ln E)

The emissions of *SO*2 and *CO*2 have a synergistic effect [54]; Sulfur dioxide and carbon dioxide emissions are highly correlated, as studied by Agee et al. (2014) [55]. As a result, it is both feasible and necessary to consider the coordinated treatment of atmospheric pollutants and greenhouse gases. Therefore, this paper will use enterprise SO2 emissions to be a substitute for carbon emissions.

In addition to this, this article employs BARTIk decomposition to break down industry-level carbon emissions into enterprise-level carbon emissions [56]as a proxy variable for enterprises carbon emissions to support our argument. Enterprise carbon dioxide emissions are approximately estimated based on industry energy consumption, with the specific calculation method outlined below. Data of industry main operating costs and total industry energy consumption obtained from the China Industrial Economic Statistics Yearbook and the China Energy Statistics Yearbook. respectively. The conversion coefficient of carbon dioxide per ton of standard coal is set at 2.493 refering to the carbon dioxide calculation standard of the Xiamen Energy Conservation Center.


$$enterprise\;carbon\;dioxide\;emissions=\frac{enterprise\;main\;operating\;costs}{industry\;main\;operating\;costs}\times total\;industry\;energy\;consumption\times carbon\;dioxide\;calculation\;standard$$ (2)

Both of these two variables have been logarithmically transformed.

#### 4.2.2 Explanatory variable: Digital Inclusive Finance (DIFI)

This paper adopts the Digital Inclusive Finance Index of Peking University to measure the development of digital inclusive finance in each prefecture-level city, and uses its sub-dimensions of total digital inclusive finance index, which are the breadth of coverage, depth of use, and digitization, for further analysis.

#### 4.2.3. Other variables

1. Technology level (*TEC*), expressed as the ratio of regional expenditure on science and technology to the general public budget. Digital inclusive finance lowers the financing threshold and broadens the financing channels to provide financial support for enterprises to adopt clean technology production, efficient and clean production, and achieve carbon emission reduction [57,58].
2. Environmental protection investment(*lninput*), which is measured by taking the logarithm of the total investment in environmental pollution control [59]
3. Financing constrain(*fincon*), which is measured by dividing interest expense by fixed assets [60].

#### 4.2.4 Control variables

Based on existing literature [24,58,61], the following control variables were selected (1) economic development level (*lngdp*), measured by logarithm of regional GDP per capita. (2) employee income (*lnwage*), measured by the average wage of employees in the region. (3) Industrial structure(*STR*), measured by the ratio of the secondary industry output value to the regional GDP (4) Total enterprise output (*lnoutput*), measured by the logarithm of the current year price of total enterprise output (5) Enterprise profitability (*roa*), measured by the total enterprise profit divided by average total assets. The first three are macro-level control variables and the last two are micro-level control variables.

### 4.3 Data sources

Most of the existing studies on digital financial inclusion use provincial-level panel data; while this paper uses enterprise-level data for analysis. The sample selected in this paper is industrial enterprises, and the time interval is set to be 2011–2015 considering the availability and completeness of the data at the enterprise level and the prefecture-level city level after matching. All the data at the enterprise level are obtained from the database of Chinese industrial enterprises and the database of China green development in China Microeconomic Database Query System; Carbon emission Accounts&Datasets [56,62–64]; the data of Peking University Digital Inclusive Finance Index and its three sub-dimensions indices are derived from the Peking University Digital Inclusive Finance Index [65]; other data come from the China Urban Statistical Yearbook, China Environmental Statistical Yearbook, China Regional Economic Statistical Yearbook, China Industrial Economic Statistics Yearbook and China Energy Statistics Yearbook in previous years. The remaining sample size of enterprises after removing missing values is 18.802, matching a total of 247 prefecture-level cities. Descriptive statistics of key variables are shown in Table 1. The typical province in our sample has the digital inclusive finance score equal to 76.19, with the minimum and maximum at 23.88 and 231.13.The statistics are largely consistent with that of prior literature(i.e.).Of relevance for quantify the firm’s carbon footprint, we observe that a fairly rich variation in two proxy variables of enterprise’ carbon emissions, ranging from zero to 15.76 and 0.56 to 11.75 respectively.

**Table 1:** Descriptive statistics.

Variables	Symbols	Definitions	Sample size	Mean	Std. Dev	Min.	Max.
Digital Inclusive Finance index	DIFI	Digital Inclusive Finance Index of prefecture-level city	18802	76.19	21.493	23.88	231.13
Enterprises’ carbon emissions	*lnE*	The logarithm of industrial emissions of sulfur dioxide(*lnSO*2)	18802	8.69	4.720	0	15.76
		See Eq (3) (*lnCO*2)	18802	6.56	3.281	0.56	11.75
Breadth of coverage of DIFI	*coverage*	Sub-dimension of DIFI	18802	74.21	24.287	4.49	217.15
Depth of use of DIFI	*usage*	Sub-dimension of DIFI	18802	75.67	25.163	12.49	223.28
Digitization of DIFI	*digitization*	Sub-dimension of DIFI	18802	76.28	28.965	2.700	262.69
Employee income	ln *wage*	The logarithm of average wage of employees in the region	18802	12.170	0.211	10.293	14.89
Economic development level	ln*gdp*	The logarithm of regional GDP per capita	18802	11.271	0.452	9.250	13.76
Industrial structure	*STR*	The ratio of the secondary industry output value to the regional GDP	18802	0.371	0.181	0.141	0.68
Enterprise profitability	*roa*	The total enterprise profit divided by average total assets	18802	0.121	0.290	-3.391	5.58
Total enterprise output	ln*output*	The logarithm of the current year price of total enterprise output	18802	11.271	1.956	0.701	17.79
Technology level	*TEC*	The ratio of regional expenditure on science and technology to the general public budget.	18802	0.02	0.012	0.0015	0.0690
Environmental protection investment	ln*input*	The logarithm of the total investment in environmental pollution control	17661	6.782	0.569	3.276	7.67
Financing constrain	f*incon*	Interest expense divided by fixed assets	18557	0.0752	0.3549	-0.2367	28.3537

## 5 Empirical results and analysis

### 5.1 Baseline regression

**A** Hausman test before regression yielded a chi-square of 102.18 and a p-value of 0, indicating that the original hypothesis was significantly rejected and a fixed effects model should be used.

Table 2 reports the regression results of the impact of digital inclusive finance on the efficiency of enterprises’ carbon emission reduction based on fixed effects. By accounting for various control variables, including macroeconomic indicators and firm-level characteristics, our analysis provides a more nuanced understanding of the impact of digital finance on environmental outcomes. Column (1) and (2) show the results when variable lnSO2 was used to substitute for lnE, and Column (3) and (4) show the results when lnCO2 was used as a proxy variable. Column (1) and (2); (3) and (4) gradually add macro control variables and micro control variables, the carbon emission reduction effect of digital inclusive finance remains significant, and Column (2) shows the logarithmic value of enterprise carbon emissions decreases by 1.62% for every one point increase in the level of digital inclusive finance; Column (4) suggests the logarithmic value of enterprise carbon emissions decreases by 1.82% for every one point increase in the level of digital inclusive finance,. The consistent and significant effect of digital inclusive finance on carbon emission reduction underscores its potential as a key driver of environmental sustainability, and indicate that the development of digital inclusive finance significantly reduces enterprise carbon emissions and promotes the improvement of enterprise carbon emission reduction efficiency, which can validate hypothesis 1. Our estimate is largely consistent with previous studies on the impact of digital inclusive finance. For instance, Sun, L. et al. (2022) found that digital inclusive finance has a positive effect on agricultural carbon emissions [14]. They demonstrated that for every percentage point increase in the level of DIF, there was a corresponding 0.207 percentage point increase in the efficiency of agricultural carbon emissions.

**Table 2:** Baseline regression results.

	(1)	(2)	(3)	(4)
VARIABLES	lnSO2	lnSO2	lnCO2	lnCO2
DIFI	-0.0167***	-0.0162***	-0.0180***	-0.0182***
	(0.18)	(0.17)	(0.03)	(0.01)
lngdp	-0.1768*	-0.1872*	0.8529*	0.8528*
	(0.19)	(0.17)	(0.58)	(0.58)
lnwage	-0.1322	-0.1465	1.9873***	1.9825*
	(0.03)	(0.03)	(0.28)	(0.24)
STR	0.0834**	0.0876**	0.8726***	0.8710***
	(0.07)	(0.08)	(0.13)	(0.14)
lnoutput		0.0783*		0.3682
		(0.06)		(0.19)
roa		0.1320*		0.7681*
		(0.06)		(0.28)
Year fixed effects	YES	YES	YES	YES
Individual fixed effects	YES	YES	YES	YES
Constant	18.59***	19.69***	-26.27***	-28.87***
	(7.70)	(7.58)	(3.87)	(4.12)
Observations	18,802	18,802	18,802	18,802
R-squared	0.95	0.95	0.67	0.67

Robust standard errors in parentheses.

*** p<0.01

** p<0.05

* p<0.1.

### 5.2 Endogeneity test: Instrumental variables estimation

In order to solve the endogeneity problem caused by omitted variables, this paper adopts the two-way "firm-year" fixed-effects model and instrumental variables method for regression.

Considering the two-way causality: on the one hand, digital inclusive finance provides financial support to enterprises by lowering the financing threshold and expanding their financing channels, thus supporting them to improve their production technology and further improve their production efficiency so that the carbon emissions can be reduced; on the other hand, the carbon emissions of enterprises can also influence the development of regional digital inclusive finance, because regions with high carbon emissions have a stronger desire to use digital inclusive finance to promote carbon emission reduction. This is because that regions with high carbon emissions have a stronger intention to utilize digital inclusion finance in promoting carbon emission reduction for the reason that there exist a pressing need to address environmental challenges and meet emission reduction targets in areas with pronounced carbon emissions. Consequently, local authorities, business nad stakeholders tend to be more proactive in exploring innovative financial solutions, and this leads to the development of digital inclusion finance in the region.

As illustrated above, we may face a problem of endogeneity, meaning that there may be unobserved factors that affect both digital inclusive finance and carbon emissions. To establish causality, we need to find two instrumental variable that affects digital inclusive finance but is not directly related to carbon emissions. This allows us to use the instrumental variables to isolate the exogenous variation in digital inclusive finance and estimate its causal effect on carbon emissions. In this case, we refer to Nunn, N., et al (2014) and use the cross product term of "distance from city to Hangzhou" and "the mean value of digital inclusive finance index outside local area" as the instrumental variable 1 (IV1), and "Number of post offices at the end of the year 1984" and "the mean value of digital inclusive finance index outside local area" as the instrumental variable 2 (IV2). The results are presented in Tables 3 and 4 and similar as in Table 2.

**Table 3:** Endogeneity test(1).

	lnE = lnSO2		lnE = lnCO2	
	(1)	(2)	(3)	(4)
	first	second	first	second
VARIABLES	DIFI	lnSO2	DIFI	lnCO2
DIFI		-0.0827***		-0.0109**
		(-3.16)		(-1.86)
IV1	0.0815***		0.1986***	
	(4.28)		(2.79)	
lngdp	4.0651*	-0.8409	-0.9861*	0.2045***
	(1.17)	(-1.67)	(-1.91)	(4.17)
lnwage	-1.9710**	-0.368*	-1.2062***	0.6782*
	(-3.29)	(-3.06)	(-1.78)	(2.06)
STR	2.2330***	0.1657**	1.9870**	-0.3752***
	(3.16)	(1.55)	(2.57)	(-2.62)
lnoutput	0.1324	0.1661*	0.2973***	-0.0917**
	(1.82)	(3.76)	(1.29)	(-3.64)
roa	-0.3250	0.0786	-0.1987	0.3986***
	(-0.87)	(0.69)	(-0.76)	(0.87)
N	18,013	18,013	18,013	18,013
F	58.46	4.83	23.67	32.56
P	0.0019	0.0000	0.0000	0.0000

t statistics in parentheses.

*** p<0.001

** p<0.01

* p<0.05.

**Table 4:** Endogeneity test(2).

	lnE = lnSO2		lnE = lnCO2	
	(1)	(2)	(3)	(4)
	first	second	first	second
VARIABLES	DIFI	lnSO2	DIFI	lnCO2
DIFI		-0.0211***		-0.0379**
		(-1.72)		(-1.27)
IV2	-0.0616***		-0.2862*	
	(-19.30)		(-33.94)	
lngdp	2.69**	-0.0230***	1.6234**	0.9839**
	(13.24)	(-2.80)	(1.27)	(3.74)
lnwage	3.2801**	0.0017	-1.8634***	-0.7963***
	(14.00)	(0.10)	(-2.23)	(-0.89)
STR	0.8907***	-0.1318*	0.1863*	-0.0972***
	(4.63)	(-6.05)	(2.65)	(-4.68)
lnoutout	-0.6319**	0.3652***	0.0223***	-0.0790***
	(-2.48)	(1.08)	(3.39)	(-2.37)
roa	-2.4801***	-0.0925**	-0.3843	0.3973**
	(-3.76)	(-3.06)	(-2.36)	(3.73)
N	17,696	17,696	17,696	17,696
F	786.17	121.64	265.16	189.79
P	0.0000	0.0000	0.0000	0.0000

t statistics in parentheses.

*** p<0.001

** p<0.01

* p<0.05.

Column (1) and (3) of Tables 3 and 4 report the F-value of first-stage estimation, which passes the weak instrumental variable test; P value present the results of the under-identification test. The results in Column (2) and (4) of Tables 3 and 4 indicate that digital inclusive finance still has a significant negative impact on enterprises carbon emissions, even when considering endogeneity problem. These results are consistent with the baseline regression results and thereby further verify hypothesis 1.

### 5.3 Heterogeneity analysis

#### 5.3.1 Regional heterogeneity analysis

There are regional differences in the impact of digital inclusive finance on enterprises’ carbon emissions. In this paper, the sample is further divided into regions east of the Hu Huanyong line and regions west of the Hu Huanyong line using the Hu Huanyong line as the boundary to examine the regional heterogeneity of digital inclusive finance on enterprises’ carbon emissions [66]. The Hu Huanyong line divides China into two regions, southeast and northwest, distinct human and natural environments exacerbating the uneven development of these two regions. The test results are presented in Table 5 and it found that the regression coefficient of digital inclusive finance is significantly negative in the area east of the Hu Huanyong line, suggesting that the development of digital inclusive finance has a significant effect on reducing enterprises’ carbon emissions in the area east of the Huanyong line; the result is not significant or only significant at 5% level in the area west of the Huanyong line,. Research of Bu,L. et al. and Wang, J et al. affirm the potential of the digital economy to break through the Hu Huanyong line and alleviate the imbalance in China’s economic development although the development pattern of the digital economy has not yet broken through the Hu Huanyong line.

**Table 5:** Regional heterogeneity analysis.

	lnE = lnSO2		lnE = lnCO2	
	(1)	(2)	(3)	(4)
VARIABLES	East	West	East	West
DIFI	-0.7621***	0.0718	-0.1972***	0.1280*
	(-2.38)	(-0.87)	(-2.99)	(0.97)
lngdp	-0.8676	2.9821	0.1862***	-1.9821***
	(-2.65)	(0.09)	(1.29)	(-1.29)
lnwage	-0.2098	0.9761	0.0208	-0.1282**
	(-0.97)	(1.12)	(0.89)	(-1.28)
STR	0.0876*	0.7619	-0.7821***	0.4723***
	(0.98)	(0.20)	(-0.96)	(0.98)
lnoutput	0.0218	0.8972	-0.2042*	0.2981
	(0.87)	(0.28)	(-2.01)	(1.61)
roa	0.1872	1.1876	0.0972***	0.7820***
	(0.18)	(1.29)	(2.98)	(3.87)
Constant	17.89**	-36.87	-2.86*	18.27***
	(1.22)	(-0.18)	(-2.32)	(2.86)
N	16,855	1,947	16,855	1,947
R-sq	0.95	0.95	0.99	0.98
Adj. R-sq	0.91	0.90	0.99	0.97

t statistics in parentheses.

*** p<0.001

** p<0.01

* p<0.05.

The geographic specificity of the DIFI effect reflects the substantial variation in local development across regions. For instance, this may be related to the fact that the level of technological development in the northwest is lagging behind that in the southeast, moreover the degree of infrastructure construction is low. Most of the headquarters and business activities of Internet financial enterprises are unfolded in the eastern region, and product and service innovations in the traditional financial industry are also basically piloted from the eastern region, moreover, the degree of marketization of capital factors in the eastern region is significantly higher than that in the central and western regions, so the eastern region has a better external environment in promoting digital financial inclusion, which lead to the limited extent of the role played by digital inclusive finance [66–68]. Therefore, compare to the eastern region,digital inclusive finance is restricted by geographical location and has limited impact on enterprises’ carbon emission reduction in the west of the line.Hypothesis 2 was verified.

#### 5.3.2 Enterprise ownership heterogeneity

The influence of digital inclusive finance on enterprise carbon emissions exhibits heterogeneity in enterprise ownership. To explore the effect of digital inclusive finance on carbon emission reduction for different types of enterprises, enterprises are divided into state-owned enterprises (SOEs) and non-state-owned enterprises (non-SOEs) according to their ownership to examine the heterogeneity effect. The regression results presented in Table 6 show that when lnSO2 was used as dependent variable, digital inclusive finance’s carbon emission reduction effect on state-owned enterprises is not significant, while the carbon emission reduction effect of digital inclusive finance on non-state-owned enterprises is significant at the 0.1% level with the logarithm of carbon emission of non-state-owned enterprises decreases by 2.0% for every one point increase in the level of digital inclusive finance; meanwhile,when lnCO2 was used as dependent variable, carbon emission reduction effect on both state-owned and none-state-owned enterprises is significant, but the significant level betrays a difference——5% and 0.1% significant level respectively. This may be attributable to the difference in access to traditional financial services between SOEs and non-SOEs.Traditional finance has already provided sufficient financial support for SOEs, therefore the diminishing marginal benefit of digital inclusive finance for SOEs limited the role of digital inclusive finance for such enterprises; meanwhile, non-SOEs which have been "left out" by traditional finance rely more on digital finance for carbon emission reduction. Therefore, the impact of digital inclusive finance on non-SOEs is more obvious and the carbon emission reduction effect is more significant. In addition, it may also be related to the differences between the internal governance structures of SOEs and non-SOEs, as well as the sensitivity and responsiveness of enterprise strategic adjustments to national policies. The economic bases of state-owned and non-state-owned enterprises are different, thus, the carbon emission reduction effect of digital inclusive finance is different.

**Table 6:** Enterprise ownership heterogeneity.

	lnE = lnSO2		lnE = lnCO2	
	(1)	(2)	(3)	(4)
VARIABLES	SOEs	Non-SOEs	SOEs	Non-SOEs
DIFI	-0.1392	-0.0201***	-0.0072*	-0.0275***
	(-1.37)	(-1.65)	(-1.56)	(-2.65)
lngdp	2.4602	-0.0128*	-0.1065	0.0265*
	(1.25)	(-0.78)	(-0.18)	(1.91)
lnwage	-0.3876*	-0.3862	-0.6582	0.1973**
	(-1.56)	(-0.11)	(-0.62)	(0.27)
STR	0.0178*	0.0431*	-0.2863*	-0.0836***
	(0.36)	(3.19)	(-1.24)	(-4.37)
lnoutput	0.5791*	0.0608	0.0297	-0.2750***
	(3.25)	(1.05)	(1.27)	(-3.46)
roa	-1.8601*	0.2060	0.1982***	0.0782***
	(-2.56)	(0.93)	(1.34)	(3.46)
Constant	9.43	17.07**	9.56**	-7.82**
	(0.28)	(1.83)	(1.47)	(-0.46)
N	3,761	15,041	3,761	15,041
R-sq	0.97	0.95	1.00	1.00
Adj. R-sq	0.92	0.93	0.99	0.99

t statistics in parentheses.

*** p<0.001

** p<0.01

* p<0.05.

The results could be a reflection of the differences between SOE and non-state-owned enterprises. For example, prior literature suggest that SOEs may have different funding sources, which can affect their business strategies and operations [69]. It is also possible that the differences in results are due to variations in the regulatory environment and government policies that govern SOEs and non-state-owned enterprises. Hypothesis 3 is verified.

#### 5.3.3 Analysis of different dimensional perspectives of digital inclusive finance

To further explore the effect of digital inclusive finance on carbon emission reduction of enterprises, three sub-dimensional indicators of digital inclusive finance: breadth of coverage, depth of use, and degree of digitalization were used for analysis. Breadth of coverage represents the ability of digital inclusive finance to reach customers, depth of use showing the depth of development of digital inclusive finance, and degree of digitalization reflects the convenience of digital inclusive finance. The results in Table 7 presented that the effect of the breadth of coverage of digital inclusive finance on reducing enterprises’ carbon emissions is not significant, while the results of the depth of use and the degree of digitalization are significantly negative. Although the results show some inconsistencies when considering breadth of coverage and degree of digitalization, Column (2) indicates depth of use of digital inclusive finance are contributing to the enterprises’ carbon emissions reduction performance. This may be due to the fact that expanding digital inclusive financial services to groups or regions where carbon emissions are not significantly associated would have limited contribution to carbon emissions reduction but the increase in the depth of use and level of digitization enables enterprises to obtain more diversified ways of financial resources which supports enterprises in enhancing production technology and achieving cleaner, reduced-emission production.Therefore, Hypothesis 4 is verified.

**Table 7:** Digital financial inclusion sub-dimensions.

	(1)		(2)		(3)	
VARIABLES	lnSO2	lnCO2	lnSO2	lnCO2	lnSO2	lnCO2
coverage	-0.0563	-0.0643				
	(0.12)	(0.36)				
usage			-0.2067***	-0.0378**		
			(0.04)	(0.18)		
digitization					-0.1782***	-0.3620*
					(0.17)	(0.05)
lngdp	-0.1432*	0.0167***	-0.1846*	0.4225**	-0.3452**	0.4876**
	(0.42)	(0.03)	(0.38)	(0.13)	(0.38)	(0.18)
lnwage	-0.0198	0.0198***	-0.1870	0.0123	-0.0182	0.0276
	(0.03)	(0.01)	(0.19)	(0.02)	(0.18)	(0376)
STR	0.0453**	-0.3522***	0.2983	-0.0536**	0.2387**	-0.0228*
	(0.02)	(0.02)	(0.29)	(0.06)	(0.06)	(0.0)
lnoutput	0.8789**	-0.0327***	0.0272**	-0.0264***	0.3782**	-0.0325***
	(0.08)	(0.52)	(0.26)	(0.20)	(0.43)	(0.02)
roa	0.0167	0.0152**	0.1204**	0.0212***	0.2912	0.1872***
	(0.71)	(0.01)	(0.92)	(0.03)	(0.10)	(0.12)
Constant	21.38*	-1.67***	21.75***	-1.25***	14.65*	-1.29**
	(3.71)	(0.358)	(7.513)	(0.428)	(7.416)	(0.405)
Observations	18,802	18,802	18,802	18,802	18,802	18,802
R-squared	0.96	0.99	0.95	0.99	0.94	0.99

Robust standard errors in parentheses.

*** p<0.01

** p<0.05

* p<0.1.

### 5.4 Mechanism analysis

In this paper, we refer to Zhu. F. et al.(2022) and utilizes the cross product term of the mechanism effect variable and explanatory variable to quantify the mechanism effect [38]. In the following section, the response variables are replaced with TEC* DIFI, which refers to technological progress effect; and ln input*DIFI, which represents environmental investment effect, to conduct the mechanism analysis. The coefficient α_1_ in Eq (2) shows the mechanism effect on the dependent variable ln E_it_.


$$\ln E_{it}=\alpha_{0}+\alpha_{1}{M_{it}*DIFI}_{it}+\alpha_{2}{Control}_{it}+\lambda_{t}+\mu_{i}+\varepsilon_{it}$$ (3)

#### 5.4.1 Technological progress effect

Digital inclusive finance expands the accessibility of financial services, which can provide enterprises with more financing channels, reduce their financing costs, and provide them with financial support for technological upgrading. In order to examine whether digital inclusive finance will promote enterprises’ technological progress, this paper selects the ratio of science and technology expenditure to general public budget to represent the technological progress effect (TEC).

The results in column (3) of the stepwise regression in Table 8 indicate that digital inclusive finance has a positive effect on enterprises’ technological progress and is significant at the 1% level. In other words, the development level of digital inclusive finance in the region where the enterprises are located is significantly and positively related to enterprises’ technological progress. And the progress of enterprise production technology can promote the transformation of enterprise input factors, improve enterprise production efficiency and resource utilization, promote enterprise green production,and reduce enterprise carbon emissions. Hypothesis 5 is verified.

**Table 8:** Mechanism analysis.

	(1)	(2)	(3)	(4)	(5)
VARIABLES	lnSO2	lnCO2	TEC* DIFI	lninput*DIFI	Fincon*DIFI
DIFI	-0.0173**	-0.0565***	0.0653***	0.9776***	-0.0472**
	(0.01)	(0.05)	(0.01)	(0.08)	(0.35)
lngdp	-0.6572*	0.8643**	1.7064***	2.7545***	-4.5734*
	(0.38)	(0.71)	(0.86)	(0.06)	(1.2415)
lnwage	-0.0182	0.6505***	-0.9874***	-0.0984**	0.4396*
	(0.05)	(0.42)	(0.12)	(0.34)	(0.02)
STR	0.2873**	1.1452***	0.0543**	0.1747**	-0.4750**
	(0.04)	(0.76)	(0.83)	(0.61)	(0.64)
lnoutput	0.0436**	0.0435	-0.0312	-0.0435	-0.9674**
	(0.86)	(0.08)	(0.73)	(0.05)	(0.94)
roa	0.9387*	0.8792	0.6923	0.8363***	0.2983
	(0.18)	(0.38)	(0.20)	(0.03)	(0.57)
Constant	13.65***	-23.64***	-16.32**	-5.3618**	17.3567*
	(5.27)	(1.62)	(2.67)	(1.46)	(3.23)
Year fixed effects	YES	YES	YES	YES	YES
Individual fixed effects	YES	YES	YES	YES	YES
Observations	18,802	18,802	18,802	17,661	18,557
R-squared	0.95	0.90	0.97	0.99	0.91

Robust standard errors in parentheses.

*** p<0.01

** p<0.05

* p<0.1.

Technological progress can have a significant impact on a firm’s carbon emissions. As technology advances, companies are able to develop more efficient processes and equipment, which can reduce the amount of energy and resources required to produce goods and services. This can, in turn, lead to lower carbon emissions. For instance, the use of renewable energy sources such as solar or wind power can reduce a company’s reliance on fossil fuels, which are a major source of carbon emissions. The positive effect of technological progress on firm’s carbon emission reduction has also been widely supported in the prior literature. For example, [70] explore the effects of technological progress in an international setting and show that technological progress will drive carbon emission reduction to improve significantly.

#### 5.4.2 Environmental investment

According to existing studies, digital inclusive finance can promote enterprises’ investment in environmental protection and thus promote carbon emission reduction. Digital inclusive finance can create more financing opportunities for enterprises, improve the success rate of financing, increase the funds available to enterprises, and promote enterprises to increase environmental protection investment such as pollutant treatment. Referring to Ren Y. et al. (2019), considering the availability of data, the logarithm of total regional environmental pollutant treatment investment is chosen to represent the environmental protection investment (ln Input) [59].

The results in column (4) of the stepwise regression in Table 8 indicate that the coefficient of the digital inclusive finance impact on enterprises’ environmental protection investment is positive and significant at the 1% level, suggesting that the level of development of digital inclusive finance in the region where the enterprise is located is significantly related to enterprises’ environmental protection investment, and the higher the level of development of digital inclusive finance, the higher the enterprises’ environmental protection investment. The increase in enterprises’ investment in environmental protection can increase the number of enterprises’ pollutant treating equipment, improve their pollution treatment capacity, therefore reducing their carbon emissions, which supports Hypothesis 6. The results of this study also have provided us with a better understanding of the impact of environmental investments on industrial emissions documented in the literature. By investing in more environmentally friendly practices and technologies, industries can make a significant contribution towards mitigating the effects of climate change.

Prior literature has provided extenstive evidence With regards to the relation between the mechanism variable(environmental investment) and outcome(carbon emission). For instance, has confirmed the positive role of green investments in reducing carbon emissions in 30 provinces in China from 1995 to 2017. Conceptually, businesses can reduce their overall carbon footprint by implementing more sustainable and eco-friendly practices, such as using renewable energy sources, reducing waste and emissions, and improving energy efficiency. Additionally, investing in research and development of new technologies and processes can lead to more innovative and sustainable practices, further reducing carbon emissions. Ultimately, enterprises that prioritize environmental protection and invest in sustainable practices can not only reduce their carbon emissions but also improve their reputation and competitiveness in the market.

#### 5.4.3 Financing constrain

Financing constraints pose a obstacle to the survival and development of enterprises, but financial development helps alleviate these constraints, thereby exerting a positive impact on the growth prospects of companies. The development of digital inclusive finance has presented new opportunities for enterprises’ sustainable growth. In order to examine whether digital inclusive finance will alleviate enterprises’ financing constrain, this paper selects the ratio of interest expense to fixed assets to represent the financing constrain effect (fincon).

The results in column (5) of the stepwise regression in Table 8 indicate that digital inclusive finance has a negative effect on enterprises’ financing constrain and is significant at the 5% level. In other words, the higher the level of development of digital inclusive finance, the lower the enterprises’ financing constrain.The decrease in enterprises’ financing constrain can increase cash flow to sustain research and development activities, consequently promoting innovation, technological advancement and environmental investment, therefore reducing their carbon emissions, which supports Hypothesis 7.

## 6 Conclusions and implications

This paper investigates the relationship between digital inclusive finance and enterprises’ carbon emissions using panel data of 247 prefecture-level cities and 6019 industrial enterprises in China from 2011 to 2015. Compared with the existing studies, the main feature of this paper is that it constructs a framework of the analysis, which is "digital inclusive finance—enhanced availability of financial services (to meet different levels of demand)—promotion of optimizing enterprises’ innovative inputs, market competition (including market integration)—efficient use of energy—enterprises’ carbon emission reduction". The study revealed that (1) digital inclusive finance can promote carbon emission reduction efficiency of enterprises; the results are still significant after using the cross multiplication of "distance from city to Hangzhou and the mean value of digital inclusive finance index outside Hangzhou" as the instrumental variable to perform the endogeneity test. (2) The heterogeneity test reveals that the effect of digital inclusive finance on carbon emission reduction of enterprises has regional heterogeneity, which shows that the effect of digital inclusive finance on carbon emission reduction of enterprises is significant in the regions to the east of Hu Huanyong Line, but not in the regions to the west of Huanyong Line. (3) The effect of digital inclusive finance on carbon emission reduction of enterprises of different enterprises ownership is also heterogeneous, which is reflected in the fact that the carbon emission reduction effect of digital inclusive finance being not significant for state-owned enterprises, but significant for non-state-owned enterprises such as SMEs. (4)There are sub-dimensional differences in the role of digital inclusive finance in enterprises’ carbon emission reduction, and the sub-dimension analysis of digital inclusive finance indicates that the depth of use has significant effects on reducing carbon emissions of enterprises. (5) Using the stepwise regression method, we verify that the effect of digital inclusive finance on enterprise carbon emissions can be achieved through the effect of technological progress, environmental protection investment and financing constrain.

Digital inclusive finance is the fusion of the value of financial inclusion and the underlying technology of financial technology, which is an important tool to achieve carbon emission reduction efficiency of enterprises. Based on the findings of this paper, the following recommendations are made:

First, our baseline results suggest that the availability of financial services, optimization of enterprises’ innovative inputs, and promotion of market competition and integration through digital inclusive finance can lead to efficient use of energy and reduction in carbon emissions. This highlights the importance of increasing financial support, investment in digital information technology such as internet technology, big data, cloud computing and blockchain to provide a higher level of technical force as a cornerstone for the development of digital inclusive finance and accelerate the innovation and upgrading of financial services; at the same time, we should accelerate the establishment of a platform for docking between enterprises and financial services, and encourage financial institutions to innovate financial instruments, so as to provide service supportive for financial and environmental protection and to further reduce financing transaction costs and mitigate enterprises’ financing constraints; Moreover, financial institutions should build a credit service evaluation system through big data so as to pinpoint target customers, identify customer needs, provide special financial support for enterprises willing to reduce emissions, and utilize the carbon emission reduction function of digital inclusive finance to promote enterprises in reducing carbon emissions.

Secondly, considering the regional heterogeneity of the role of digital inclusive finance in carbon emission reduction for enterprises that we show in Section 4.3.1, attention should be paid to the availability, usage and quality of financial services in less developed regions. Resources should be coordinated and deployed so that labor, material and financial resources and other relevant resources can be tilted to the western region where the development of digital inclusive finance is weaker. In the meantime, financial institutions are encouraged to develop their operational outlets to inject new impetus for high-quality economic development in the less developed western region; and digital inclusive finance should be developed in accordance with local conditions. The developed eastern region should focus on the innovation and upgrading of digital financial tools to meet the in-depth and diversified financial needs of enterprises, while the western region, where the development of digital inclusive finance is weak, should focus on the improvement of financial infrastructure and the popularization and promotion of digital financial services provided to enterprises.

Thirdly, we also find that the degree of reliance on digital inclusive finance varies among enterprises of different ownerships. The results indicate that policymakers should pay special attention to supporting non-state-owned enterprises, enriching the financing channels of state-owned enterprises and helping non-state-owned enterprises to obtain more accurate financial services.

Forth, the analysis of different dimensional perspectives of digital inclusive finance in Section 4.3 suggests that the construction of digital inclusive financial service system should be strengthened to further enhance the digitalization, coverage breadth and usage depth of digital inclusive finance, improve the construction of infrastructure related to financial and digital services, establish a complete digital financial service network, continuously expand the coverage of financial services, and further combine digital inclusive finance with enterprises’ carbon emission reduction needs effectively. Moreover, by combining the existing service network advantages of financial institutions with the technical data resource advantages of digital technology, the speed and scale of the development of digital inclusive finance can be continuously improved. This will strengthen the service capabilities and promotion role of digital inclusive finance for physical enterprises, and fully unleash the positive role of digital inclusive finance in promoting green production and living and promoting the development of a low-carbon economy.

Fifth, the implication of our mechanism analysis is that enterprises should be guided to upgrade production technology, increase investment in environmental protection, adjust their own financing structure, and make full use of multiple channels to obtain financial support. Enterprise managers should change their conservative mindset, accept new things, improve their policy sensitivity, and actively respond to the national policy of " carbon neutrality" and "digital economy", as well as establish environmental awareness, pay attention to energy saving and emission reduction. More importantly, they should take the initiative to learn and understand how to utilize digital inclusive finance efficiently to broaden financing channels and obtain sufficient financial support for improving production technology, enhancing production efficiency, and increasing environmental protection investment, and consequently achieving carbon emission reduction.
